# Towards a Computational Model of a Methane Producing Archaeum

**DOI:** 10.1155/2014/898453

**Published:** 2014-03-04

**Authors:** Joseph R. Peterson, Piyush Labhsetwar, Jeremy R. Ellermeier, Petra R. A. Kohler, Ankur Jain, Taekjip Ha, William W. Metcalf, Zaida Luthey-Schulten

**Affiliations:** ^1^Department of Chemistry, University of Illinois at Urbana Champaign, Urbana, IL 61801, USA; ^2^Center for Biophysics and Computational Biology, University of Illinois at Urbana Champaign, Urbana, IL 61801, USA; ^3^Department of Microbiology, University of Illinois at Urbana Champaign, Urbana, IL 61801, USA; ^4^Department of Physics, University of Illinois at Urbana Champaign, Urbana, IL 61801, USA

## Abstract

Progress towards a complete model of the methanogenic archaeum *Methanosarcina acetivorans* is reported. We characterized size distribution of the cells using differential interference contrast microscopy, finding them to be ellipsoidal with mean length and width of 2.9 **μ**m and 2.3 **μ**m, respectively, when grown on methanol and 30% smaller when grown on acetate. We used the single molecule pull down (SiMPull) technique to measure average copy number of the Mcr complex and ribosomes. A kinetic model for the methanogenesis pathways based on biochemical studies and recent metabolic reconstructions for several related methanogens is presented. In this model, 26 reactions in the methanogenesis pathways are coupled to a cell mass production reaction that updates enzyme concentrations. RNA expression data (RNA-seq) measured for cell cultures grown on acetate and methanol is used to estimate relative protein production per mole of ATP consumed. The model captures the experimentally observed methane production rates for cells growing on methanol and is most sensitive to the number of methyl-coenzyme-M reductase (Mcr) and methyl-tetrahydromethanopterin:coenzyme-M methyltransferase (Mtr) proteins. A draft transcriptional regulation network based on known interactions is proposed which we intend to integrate with the kinetic model to allow dynamic regulation.

## 1. Introduction

Molecular signatures of ribosomal rRNA evolution were used by Woese and his associates to establish the three primary groupings of living organisms: archaea, bacteria, and eukarya [1–6]. Although the ancestral or communal origins of these three domains remains a matter of debate, increasingly large amounts of data regarding the RNA phylogeny and molecular makeup of cells accumulated over the last several decades continue to support the division between the three primary domains [7]. Furthermore, comparative analysis of the sequences of proteins and RNA involved in translation provides strong evidence that the existence of highly developed translational machinery was a necessary condition for the emergence of cells as we know them [8, 9]. Molecular signatures in the ribosome—idiosyncrasies in its rRNA [7] and/or r-proteins characteristic of each domain of life—were locked in place at the time of evolutionary divergence, destined to become molecular fossils. As Woese postulated in his theory of genetic annealing, ancestors of the three primary groupings of organisms developed into a number of increasingly complex cell types. The various subsystems of the cell “crystallized,” that is, became refractory to lateral gene transfer with the translation apparatus probably crystallizing first.

While the rRNA phylogeny is supported by phylogenetic analysis of concatenated protein sequences of the fundamental genes in the translational machinery, the effects of lateral gene transfer (LGT) among organisms in the three domains of life are clearly seen in the aminoacyl-tRNA synthetases, a modular subsystem that charges tRNA, and helped to establish the genetic code [10–12].

As one moves beyond the information processing systems of translation and transcription, an increasing amount of LGT also extends into other cellular networks. If the early community of cells was more like a modern bacterial consortium, the cells could have cross-fed one another not only genetically but also metabolically. Every improvement in translation that increased its accuracy would have permitted new proteins to emerge, which in turn could have further developed the metabolic pathways within cells. With metabolic functions being modular in nature, these genes could be transferred laterally. Many cases are now known in which a bacterial metabolic gene occurs in one or a few archaea or vice-versa and has prompted the search for signatures in the metabolic networks that are distinctive of the archaea [13–16].

Many theories of early life argue for a reducing environment in which anaerobic organisms would likely be the first to have evolved [17]. A phylogenetic analysis of proteins that are distinctive of archaea and its main subgroups has led to hypotheses in which methanogens—anaerobic archaeal organisms that derive all of their metabolic energy by reduction of single carbon compounds to methane—feature prominently in the early evolution of life. Methanogens are phylogenetically diverse group of strict anaerobes estimated to produce a billion tonnes of methane per year [18]. They are found in niche environments including shallow and deep hydrothermal vents [19], swamps, paddy fields, land fills [20], hot-springs, and oxygen-depleted sediments beneath kelp beds [18].

The *Methanosarcineae* are the most metabolically diverse methanogens known. Only *M. acetivorans* and other archaea in the genus *Methanosarcina* use all four known metabolic pathways for methanogenesis under different growth conditions. While systems biology studies have long used *E. coli* as a model organism in understanding the response of cellular networks to changes in various environmental conditions or gene knock-outs, computational models of methanogen metabolism are just beginning to be established [21, 22]. Based in part on our own work modeling genetic switches in *E. coli* and the effects of heterogeneity in protein expression on the metabolism of large populations of bacteria [23], we present here our progress toward a comprehensive computational model of a methanogen. In doing so we have been profoundly influenced by our association with Carl Woese, who published the first genome of a methanogen, *Methanocaldococcus janaschii*, and greatly inspired our interest in characterizing both the translational and metabolic machinery of methanogenic archaea [24].

We have focused our study on *M. acetivorans* for several reasons. First, the organism can grow on three classes of substrates demonstrating use of three methanogenic pathways: (1) methylotrophic pathway, wherein the organism grows on methyl containing substrates including methanol, tri-, di-, and mono-methylamine (TMA, DMA, MMA), and methylsulfides (DMS, MMS); (2) acetoclastic pathway, wherein the organism grows on acetate; (3) carboxidotrophic pathway, wherein carbon monoxide is oxidized to acetate, formate, and methane [25–27]. Second, the genome of *M. acetivorans* has been sequenced [25], and considerable effort has been expended towards determining the regulation of gene expression of methanogenesis proteins [28]. Third, the genome exhibits considerable homology to two other well studied members of the genus *Methanosarcina*: *M. barkeri* and *M. mazei* and, therefore, a model for one will likely be easily modified to work for the others.

Developing a model of the archaeum requires characterization of its physical and biochemical properties. To that end the physical dimensions of the cells, including their length and width, were measured. Modeling also requires estimation of protein/ribosome copy numbers in single cells; the single molecule pulldown (SiMPull) technique [29]—a marriage of the conventional pull-down assay with single molecule fluorescence microscopy—was used to measure the mean copy number of two key proteins. The first protein measured was the *γ* subunit (McrG) of methyl-coenzyme-M reductase (Mcr) complex as a proxy for number of Mcr complexes, which catalyze the methane producing step of methanogenesis. Second, the ribosomal protein Rpl18p in the large subunit of ribosome was counted as a proxy for the number of ribosomes. A kinetic model for methanogenesis pathways capable of representing growth on methanol and acetate was developed using RNA-seq data and kinetic parameters from the literature. This model captures several features of comparable experimental data [30, 31]. The model further allows us to probe the sensitivity of the growth (and indirectly the methane production) on the copy number of each protein, directing further experimental study. In an effort to extend the model to simulate growth on other substrates, we compile a list of all experimentally known and hypothetical transcriptional regulatory interactions. These interactions will be used to modulate protein expression as a function of growth substrate that we can marry with the kinetic model in future.

## 2. Experimental and Computational Methods

### 2.1. Strains, Media, and Growth Conditions

*M. acetivorans* C2A strains (wild-type, WWM 889  :: *SNAP-mcrG*, and WWM890  :: *rpl18p-SNAP*) were grown in single cell morphology [48] at 37°C in high-salt (HS) medium containing either 125 mM methanol or 40 mM acetate [49]. Handling and manipulation of all strains were carried out under strict anaerobic conditions in an anaerobic glove box, using sterile anaerobic media and stocks. Solid media plates (HS medium, 1.5% agar) were used for selection of SNAP integrants in two steps: puromycin (Research Products International, Mt. Prospect, IL) at a final concentration of 2 µg/mL was used for selection of strains carrying puromycin transacetylase (*pac*) and the purine analogue 8-aza-2,6-dia-minopurine (8-ADP) (Sigma, St. Louis, MO) at a final concentration of 20 µg/mL was used for selection against the *hpt* gene [50–52]. All plates were incubated in an anaerobic intrachamber incubator [53]. Standard methods were used throughout for isolation and manipulation of plasmid DNA from *E. coli*. DNA purification was performed using appropriate kits (OmegaBio-Tek, Norcross, GA). Growth was quantified by measuring the optical density at 600 nm (OD_600_, Milton Roy Company Spectronic 21 spectrophotometer) and generation times were calculated during exponential growth.

### 2.2. Genetic Constructs in Methanosarcina Acetivorans

Genetic fusions with SNAP were made by first constructing plasmids with the SNAP gene near an *aphII* cassette flanked by NheI restriction sites. pJK1048A was used as the template for making fusions to the C-terminus of genes of interest, while pJK1047B was used for fusions to the N-terminus. DNA oligonucleotides (IDT, Iowa City, IA) with homology to the template and gene of interest were used to amplify the SNAP-aphII constructs. The Lambda Red method was then used to integrate SNAP aphII construct into specific N- or C-terminal locations [54], selecting for kanamycin resistance. The *mcrG* and *rpl18p* genes are carried on cosmids created during an *M. acetivorans* cosmid library construction previously performed in the Metcalf lab (Zhang and Metcalf, unpublished). The *aphII* allele was then excised from the cosmid by NheI restriction digest, leaving an in-frame SNAP fusion to the gene of interest. The wild type copies of the genes in question were replaced by the SNAP tagged versions using homologous recombination, as previously described [51].

### 2.3. Cell Morphology from DIC Microscopy

Cell cultures were grown into exponential phase to an OD_600_ of 0.6 and 1 mL of cultures was removed and centrifuged at 14,000 g for 5 minutes. The cell pellet obtained was resuspended in 100 *μ*L HS media without resazurin, and the cells were observed using the differential interference contrast (DIC) microscopy technique on a Zeiss LSM700 confocal microscope.

### 2.4. RNA-Seq Analysis

*M. acetivorans* C2A wild type was adapted to methanol and acetate for 33 generations. The total RNA was isolated from early exponential phase cultures (OD_600_ = 0.4) using TRIzole (Invitrogen, Carslbad, CA) and the Zymo Direct-zol RNA MiniPrep kits (Zymo Research, Irvine, CA). The RNA samples were depleted of the 16S- and 23S-rRNA through hybridization to complementary biotinylated oligonucleotides and subsequent removal with streptavidin-magnetic beads (modified from [55]). Construction of cDNA libraries and high throughput sequencing of RNA was carried out by the Roy J. Carver Biotechnology Center at University of Illinois at Urbana Champaign. All measurements were done in triplicate. The Rockhopper [56] bacterial RNA-seq analysis software was used to map RNA reads to the *M. acetivorans* genome using the default parameters with verbose output enabled. Reads per kilobase per million reads (RPKM) values from the three replicates were averaged and used in subsequent analysis.

### 2.5. SiMPull Experiments

**Figure 1:**
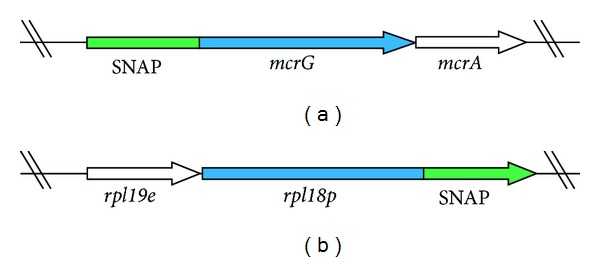
Genetic constructs showing position of SNAP relative to our protein of interest on chromosome of *M. acetivorans*. (a) N-terminal label on *mcrG* gene. (b) C-terminal label on *rpl18p* gene.

The single molecule pulldown, or SiMPull technique [57], was used to determine mean protein counts for two proteins in *M. acetivorans*. Briefly, SiMPull is a microscopy technique wherein a fluorescently labeled protein of interest is “captured” out of cell lysate by an immobilized antibody attached to a passivated microscope slide. In these experiments, the genetic SNAP-tag system (New England Biolabs (NEB), Ipswich, MA) was used for labeling either the N- or C-terminus of each protein studied (Figure 1).

Labeled mutants were grown to exponential phase and harvested at an OD_600_ of 0.6. Cell density was estimated using a Petroff-Hausser counting chamber. One milliliter of cell culture was centrifuged at 14,000 g for 5 minutes to obtain cell pellets which were subsequently lysed upon re-suspending the cells in 100 µL of the recommended SNAP labeling buffer: 50 mM Tris-Hcl (pH 7.5); 100 mM NaCl; 0.1% Tween 20; 1 mM DTT (NEB) with 1 µg DNAse. The cell lysate was then incubated with AlexaFluor 488 (NEB) at a final concentration of 10 µM at room temperature for one hour. In order to remove free dye, samples were washed three times with SNAP labeling buffer and concentrated using 10 K Amicon ultra centrifugal filters. SiMPull analysis was performed as previously described [57].

Microscope slides were coated with polyethylene glycol (PEG) which minimizes nonspecific biomolecule adsorption. Surfaces were doped with 2–5% biotin-conjugated PEG during slide preparation. The bait recruiting rabbit anti-SNAP antibody (NEB) was immobilized onto the surfaces by successively flowing in NeutrAvidin (4 µM) and a biotin conjugated antirabbit antibody (20 nM), as depicted in Figure 3(a).

Lysate was washed away and the pulled-down proteins were imaged using a prism type Total Internal Reflection Microscopy (TIRF) with excitation at 488 nm. The resulting images (see Figure 3(b)) were analyzed using custom software as described previously [29], to quantify single spots in the field of view of the microscope. A single spot may correspond to more than one fluorophore which can be discerned by the observation of multiple discrete photobleaching steps (as in the case of Rpl18p, Figure 9) indicating that results are lower bounds on the actual number of proteins.

### 2.6. Kinetic Model

RNA-seq expression data for *M. acetivorans* growing on methanol and acetate [59] provide enough parameters for a preliminary kinetic model of the methanogenesis pathways (Table 1). The model includes reactions for the methylotrophic, acetoclastic, and electron transport pathways shown in Figure 4. An additional reaction simulating biomass growth is included to the model that converts ATP created by the methanogenesis driven proton gradient into cell mass. Because 98% of carbons that come into methanogenesis leave as CH_4_ or CO_2_ [60], ATP is assumed to be a good analog for the growth of the colony. A model schematic is shown in Figure 5.

The kinetic model in Table 1 is based on the reactions from metabolic model *iMB745* [22]. The reactions are modeled as a set of coupled differential equations (ODEs) which are solved deterministically using the COPASI software [61]. Rate data for 17 of the 26 methanogenesis reactions were taken from the literature [32–38, 40–47] as reported in the BRENDA database [62]. The other 9 parameters were fit to experiments wherein a cell culture was grown on 125 mM methanol [30]. Three types of reaction mechanisms are used to model the reactions: irreversible unimolecular Michaelis-Menten, irreversible bimolecular Michaelis-Menten, and first order. In cases with more than two reactants, the two most important reactants were selected for bimolecular reaction and a constant flux reaction was added that converts the additional reactants to products at the same flux as the rate of bimolecular reaction. In bimolecular reactions, *k*_cat_ and *K*_*M*_ for both substrates were assumed to be the same. When missing from the literature, *K*_*M*_ parameters for reverse reactions were assumed to be the same as that for forward reactions (e.g., Mtd, Mch, Ftr, and Fmd/Fwd). The forward and reverse rate constant are known for Mer giving a ratio of about 6.8. This ratio was assumed for Mtd, Mch, Ftr, and Fmd/Fwd as they are in the same pathway. Because Mtr is known to be nearly at equilibrium [63], we assumed the forward and reverse rates were the same. A value of 50 s^−1^ was chosen for this reaction.

Finally, Rnf and Fpo were assumed to have similar rates to the Hdr protein as they also catalyze the motion of a similar number of ions across the cell membrane. The reactions modeled and rate constants used in the model can be found in Table 1.

A value of 15.4 grams of cell mass per mole of ATP [22, 64] was used in the biomass expression to match the stationary phase mass of a culture calculated from experimental OD_420_ measurements [31]. The rate of the cell mass reaction was set to match the approximate maximal doubling time of 8 hours known for growth on methanol. The accumulation of biomass in the model leads to an accumulation of enzymes; for each gram of biomass, 63% is assumed to be proteins (in accordance with [22]) of which some are the methanogenic enzymes that themselves catalyze growth. The results of RNA-seq experiments provide estimates for the stoichiometry of methanogenic enzymes per mole ATP. A linear relationship between methanogenic proteins and mRNA was assumed. We determined the relative mass of protein as

(1) $$\begin{array}{c} 0.63M_{\text{total}}=\sum_{i=1}^{N_{\text{genes}}}a_{i}\times m_{\text{Protein},i}, \end{array}$$

where the coefficients *a*_*i*_ are the mass fraction of *i*th protein calculated with (2). From the value of *a*_*i*_ and the molecular weight of protein *m*_protein,*i*_, the number of moles of protein per mole of ATP was determined; these values are provided in Table 2. We have

(2) $$\begin{array}{c} a_{i}=\frac{m_{\text{protein},i}}{\sum_{i=1}^{N_{\text{genes}}}m_{\text{protein},i}\times {\text{RPKM}}_{i}}. \end{array}$$

The model was solved in a 1 mL volume with an initial cell mass of 0.1 mg, calculated from the optical density at the start of growth [30, 31]. The concentrations of water and internal protons are assumed to be constant and therefore their effect on the rate constants is implicit and not explicitly modeled. The concentration of extracellular protons was initially set to physiological pH of 7 and protons are modeled explicitly in the ATP synthase reaction. This reduces the complexity of most of the reactions to either one or two substrate Michaelis-Menten kinetics. Initial concentrations of ATP, ADP, and P_i_ were set to physiological concentrations of 10, 1, and 10 mM, respectively [47]. Intermediate energy carriers (CoB, CoM, ferredoxin, etc.) initial concentrations were assumed to be 0.009 mM, which was calculated from the measured value of 474 nmol/g protein measured for coenzyme F_420_ in *M. barkeri* grown on methanol [65].

### 2.7. Transcriptional Model

A putative model of transcriptional regulation was constructed using experimental data and inferred regulatory interactions based on gene annotation and sequence homology with proteins known to be regulated in other archaea. Two different models were developed: the first involving only direct interactions and the second involving indirect and hypothetical interactions. The direct interactions model was based on experimental evidence of actual binding of the activator/repressor to the promoter region causing up/downregulation of target gene. In addition, genes that showed differential expression and contained the known promoter region, were included in the direct model. The indirect interaction model includes interactions reported in the literature where proteins were differentially expressed under different growth conditions or when expression correlated with a regulator that is differentially expressed, but no direct evidence for the interaction exists. Strength of interactions in the direct and indirect models were taken from the literature; when the transcriptional regulator was overexpressed, the strength of interactions was normalized by the overexpression level. A full enumeration of the literature used to develop these transcriptional regulation models is reported in Section 3.5.

## 3. Results and Discussion

### 3.1. Cell Characterization

DIC images of methanol and acetate grown cells were obtained and analyzed in order to quantify their physical dimensions. As seen in Figure 7(a), DIC microscopy yields enhanced contrast images by taking advantage of a gradient in optical path length between beams of light passing through adjacent points in the illuminated sample. The enhanced contrast is directional and appears strongest along the shear vector. No contrast occurs perpendicular to the shear vector, which can make the demarcation of cell boundaries difficult. A Hilbert transform has been used in the past with DIC microscopy in order to aid in image segmentation [66]. Custom Matlab scripts were developed to normalize and apply a Hilbert transform to the DIC images. The transformed image shows clearer boundaries around the imaged cells (Figure 7(b)). The CellProfiler software was used to identify cell boundaries [67] and measure the cells' dimensions.  Figure 6 shows the distributions of lengths and widths obtained from approximately 10,000 identified cells. The mean length and width observed were 2.9 µm and 2.3 µm for methanol grown cells, while for acetate grown cell they were 2.3 µm and 1.7 µm, respectively. Assuming the cells to be ellipsoid in shape, volume of a methanol grown cell would be approximately 9 fl and that for acetate is approximately 4 fl. Cells have mean aspect ratios of 1.27 for methanol grown cells and 1.33 for acetate grown cells.

### 3.2. SiMPull Measurements

**Figure 2:**
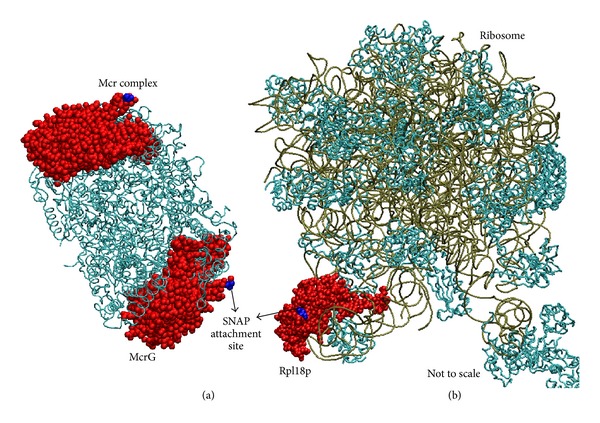
(a) The Mcr complex from *M. barkeri* (1E6Y [58]) with the McrG subunit shown in red with the SNAP attachment site shown in blue. (b) The large subunit of an archaeal ribosome (*Haloarcula marismortui*, 4HUB) showing the L18p subunit in red and the C-terminus where the SNAP is attached in blue. These suggest that the position of SNAP is on the outer part of the complexes, which enables capture by the SNAP antibody.

**Figure 3:**
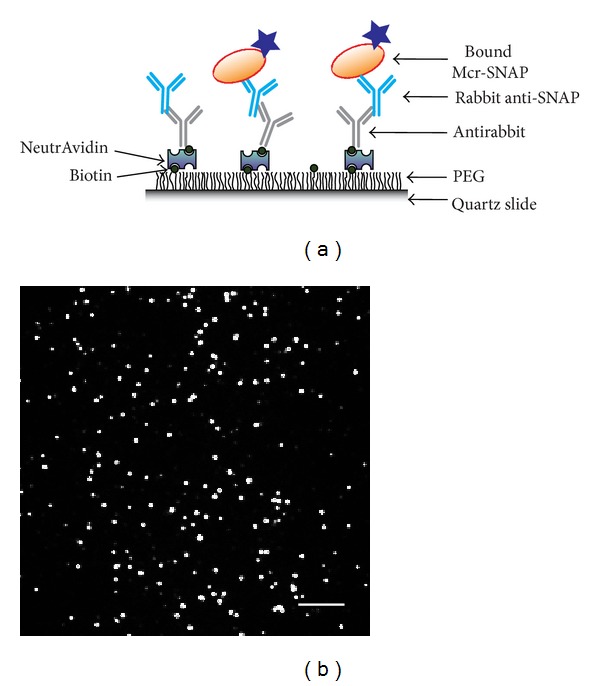
SiMPull experiments for protein count measurement. (a) Anti-SNAP antibody immobilized on a microscope slide using biotin. The antirabbit antibody captures SNAP labeled McrG. (b) A TIRF microscopy image obtained for captured McrG, where each spot corresponds to at least one immobilized protein.

**Figure 4:**
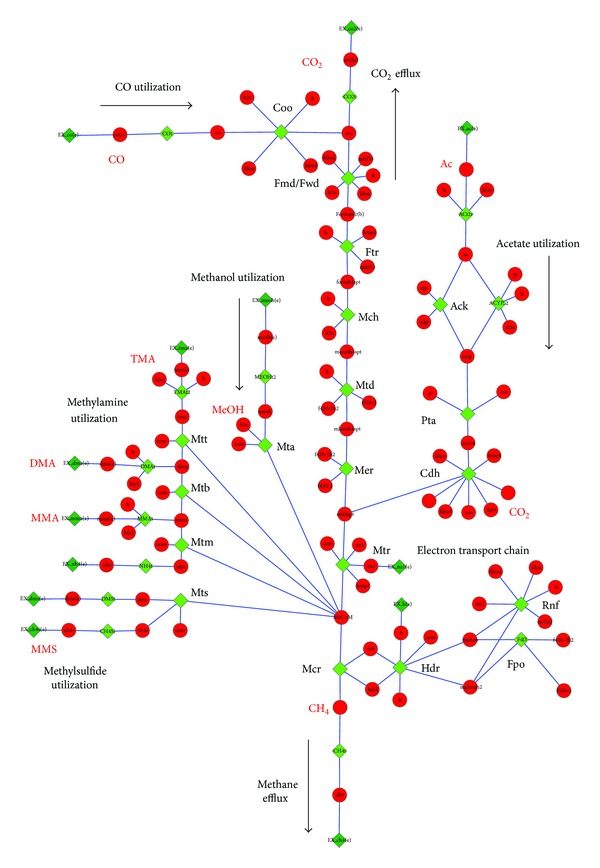
Methanogenesis pathways in metabolic map of *M. acetivorans* [22]. Enzymes and metabolites are depicted as nodes while reactions are depicted as edges between these nodes. Enzymes that catalyse reactions are shown as green diamonds and metabolites are shown as red circles. Enzyme names are in black and select metabolite names are in red.

**Figure 5:**
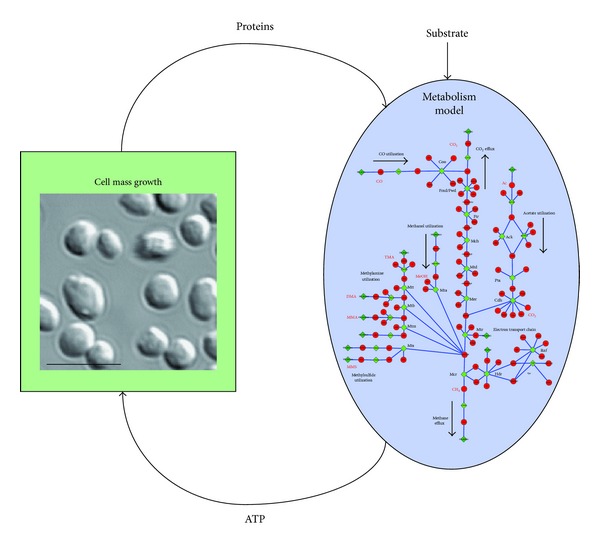
A schematic of the kinetic model. Flux of ATP from the methanogenesis pathway (see Figure 4) feeds into a cell growth term that updates protein numbers used by the kinetic model, simulating the growth when fed on a certain substrate. The inset in the cell mass growth expression is from DIC microscopy with a 5 µm scale bar.

**Figure 6:**
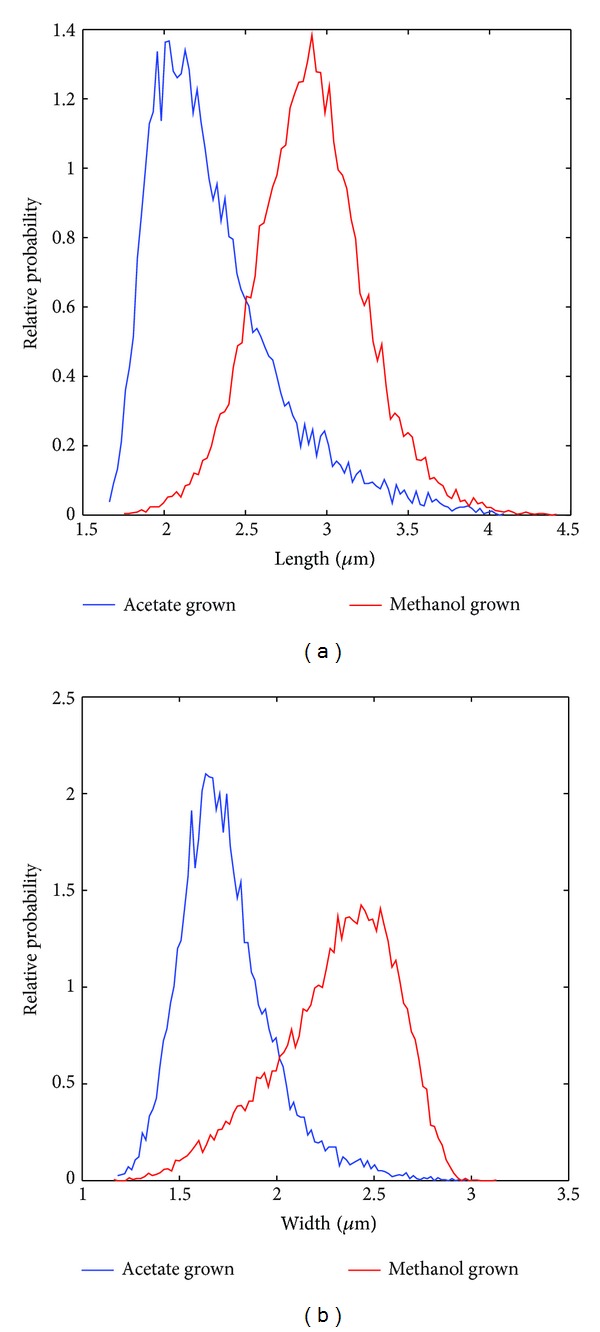
Distributions of (a) lengths and (b) widths of single *M. acetivorans* cells grown on methanol (red) and acetate (blue) as determined by DIC microscopy and image analysis. Data corresponds to approximately 10,000 cells.

**Figure 7:**
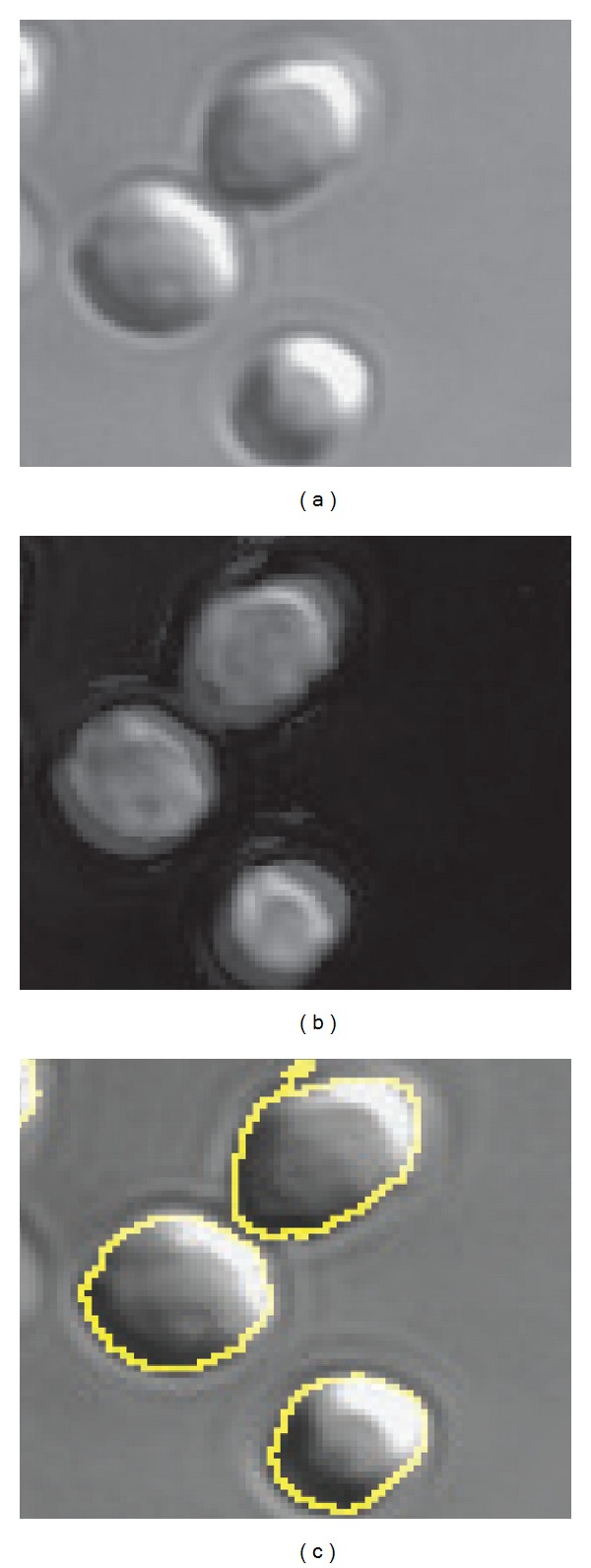
(a) DIC images of *Methanosarcina acetivorans* grown on methanol. (b) After applying Hilbert transform to DIC images and adding them to the original image, the boundaries of the cells become clearer. (c) CellProfiler is used to perform segmentation on images in (b). Here identified cell boundaries are superimposed onto original DIC image to illustrate segmentation.

**Figure 8:**
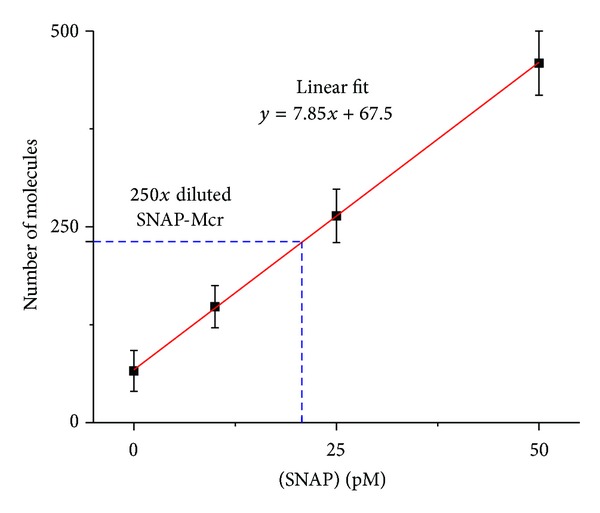
Calibration curve for SiMPull experiments that provides a mapping between protein concentration in cell lysate and number of spots observed on the slide.

**Figure 9:**
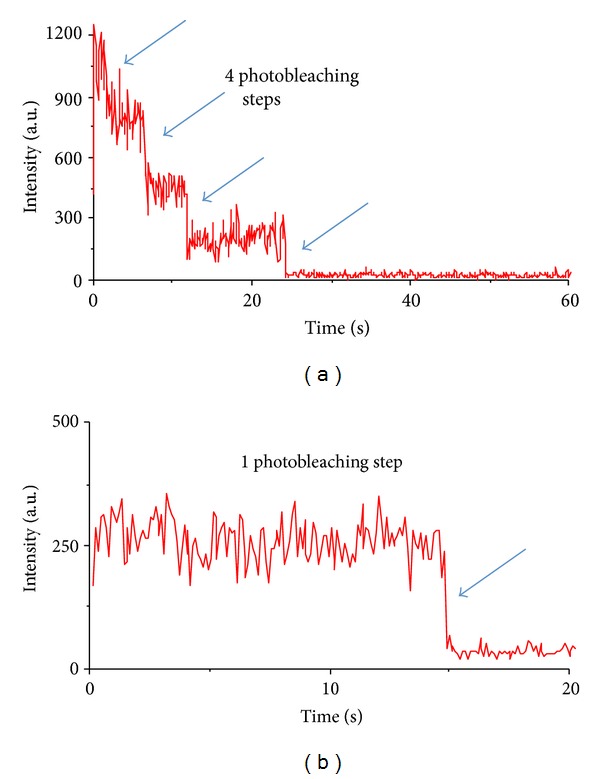
Multiple photobleaching steps in SiMPull experiments with rpl18p-SNAP strains (a) indicate multiple immobilized proteins as compared to pure SNAP protein which shows only single photobleaching step (b).

SiMPull was used to measure the mean copy numbers of two proteins integral to the growth and physiology of methanogens. The first is the *γ* subunit of the methyl-coenzyme M-reductase (Mcr) complex. This complex is a lynchpin in the metabolic network, catalyzing the last step of methanogenesis that produces methane. The second is Rpl18p, a ribosomal protein counted as proxy for the ribosome, the protein producing machinery of the cell. We have inserted SNAP tags at the N-terminus of the *mcrG* gene and at the C-terminus of the *rpl18p* gene (Figure 1). The fusion of SNAP at the C-terminus of Rpl18P exposes it to the outside of the ribosome enabling the immobilized anti-SNAP proteins to capture whole ribosomes during the SiMPull assay (see Figure 2). Using these calibration curves (Figure 8), along with estimates of cell density prior to lysing from cell counting experiments, copy numbers of Mcr and ribosomes per cell were obtained (Table 3). Mcr numbers agree qualitatively with a recent study where Mcr was imaged on TEM immunocytochemistry techniques [68].

### 3.3. RNA-Seq Experiments

RNA sequencing experiments were performed in order to elucidate the differential expression of methanogenic enzymes on different growth substrates. Comparison of the ratio of mRNA expression on methanol-grown cells to that of acetate-grown cells shows good agreement with the results of quantitative reverse transcriptase polymerase chain reaction (qrtPCR) data previously reported [69].

However, two discrepancies are observed. The first is that Hdr, which was found to be more highly expressed in methanol-grown cells than in acetate-grown cells, was previously reported to be more highly expressed under acetate growth conditions. This is likely attributable to experimental noise as in both cases the expression ratios are close to 1. The other difference is more pronounced; expression of ATP synthase was found to be 3 times higher on acetate in our experiments, whereas the previously reported results indicate only a two fold enhancement. In spite of this, expression of methanogenic proteins generally agree with previous reports [59, 69], and, importantly, our experiments were run in triplicate and, therefore, offer greater confidence in our results in addition to some means of error estimation.

Protein numbers, computed using the assumption that they are linearly proportional to mRNA number from RNA-seq, were compared with experimentally measured ones from SiMPull. From [26], we know that OD_420_ of 1 corresponds to 0.41 ± 0.07 mg dry mass per mL of culture grown on CO. Assuming 63% of this mass being protein and Mcr having 1.2% mass fraction in the proteome, we obtain 3.1 ± 0.5 µg of Mcr per mL of culture or 10.3 ± 1.6 picomoles per mL of culture (molecular weight of Mcr = 300 kDa [70]). Using cell density of 500 million cells per mL of culture at OD_420_ of 1 (data not shown), we obtain around 12,400 ± 2,000 copies of Mcr per cell grown in methanol and approximately 6,000 for cells grown on CO estimated due to size differences. SiMPull measurements for Mcr in methanol grown cells ranged from 273 to 1320 copies per cell (see Table 3).

### 3.4. Kinetic Model

**Figure 10:**
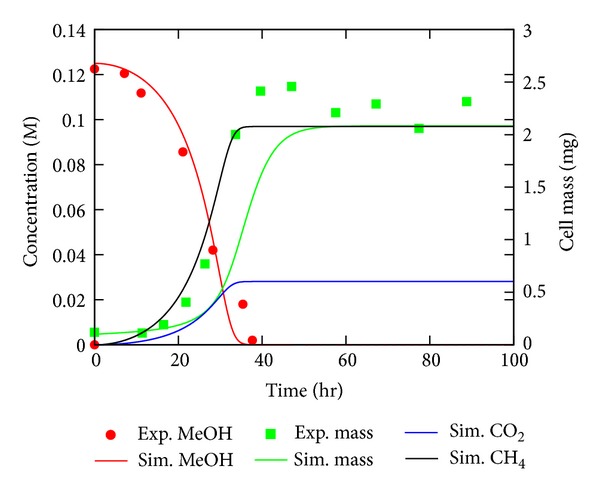
Results of the kinetic model. Comparison of the kinetic model for growth of *M. acetivorans* culture on 125 mM methanol to the experiment to which it was fit [30]. Lines indicate model results while symbols indicate experimental measurements.

A kinetic model of the methanogenesis pathways in* M. acetivorans* with single-reaction resolution was developed. The model was fit to cell culture growth experimental results reported previously [30] wherein the methanol consumption rate and OD_420_ of cell culture were studied over time. A comparison of the model fit, seen in Figure 10, demonstrates that the chosen rate parameters capture the methanol behavior within 10%. The cell mass in the culture was calculated from OD_420_ traces from [30] using the calibration point of 0.41 ± 0.07 mg/mL at OD_420_ 1.0. At maximum growth rate, the model predicts methane formation of 565 nmol/mL × min (slope of simulated curve in Figure 10) compared with 372 ± 69 nmol/mL × min measured experimentally [31]. The model correctly predicts the mass of the cells in culture (within 10%), and it captures the 3 : 1 methane to CO_2_ efflux ratio that is necessary for the correct redox intermediate behaviors. Using a stoichiometry of 3.5 protons per ATP, as measured in some experiments for other organisms [71], would make the modeled cell mass growth exactly match the experiments.

**Figure 11:**
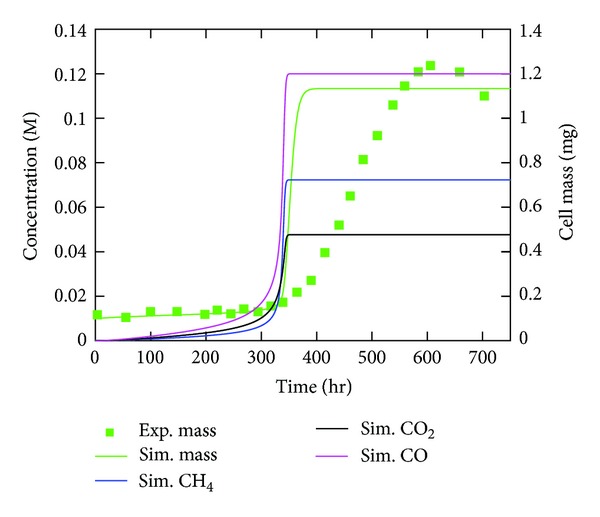
Results of the kinetic model. Comparison of the kinetic model for growth of *M. acetivorans* culture on 120 mM acetate to the experiment [31]. Lines indicate model results while symbols indicate experimental measurements.

Acetate growth uses a different methanogenesis pathway and is a good test for the rate constants. Using RNA expression values of proteins in acetate-grown cells and the same kinetic parameters obtained from the methanol fit, the results along with experimental results for cells grown in 120 mM acetate [31], shown in Figure 11, were obtained. Cell mass entering stationary phase is of the right level, but the rate of growth is much too high. The model predicts a significant buildup of carbon monoxide, which should be converted to CO_2_ as that step produces more electrons used to drive protons across the membrane. The methane production rate was determined to be 269 nmol/mL × min, which is less than the rate of methane production on methanol but is quite a bit higher than experimental measurements of 82 ± 31 nmol/mL × min [31].

This model represents a powerful tool for its ability to be used in testing the sensitivity of cell growth to model parameters such as enzyme copy numbers and rate constants. Moreover, because the growth rate can be thought of as a proxy for the amount of methane produced, understanding its sensitivity to enzyme expression is interesting from a biofuels perspective. The relative sensitivity, *s*, is calculated using the standard expression:

(3) $$\begin{array}{c} s=\frac{x}{Y\left(x\right)}\frac{\partial Y\left(x\right)}{\partial x}, \end{array}$$

where *Y* is the observable (e.g., growth rate) and *x* is the parameter (e.g., enzyme copy number). Performing this analysis for methanol growth shows that cellular growth rate is most sensitive to the copy number of Mcr and, in order from greatest to least sensitivity, the copy numbers of Mtr, Rnf, Fpo, MtaCBA2, HdrDE, Mer, and MtaCBA1. This suggests that growth rate is most dependent on the rate at which methyl-coenzyme M can be reduced to methane. These results also indicate that growth rate depends on the equilibrium of species at the branching point that directs substrates either to CO_2_ or to methane via the Mtr reaction. This is in line with the fact that the acetoclastic pathway is highly downregulated during growth on methyl substrates, driving flux through that reaction in the reverse direction. In addition, the rate at which protons are pumped across the membrane and the intermediates regenerated (via Hdr and Rnf) effect the rate significantly. Finally, it is of no surprise to see that the rate of methane production is strongly dependent on the rate at which methanol is brought into the methanogenesis pathway as demonstrated by the dependence on Mta proteins.

Examination of the sensitivity of growth rate to various enzyme copy numbers under acetate-growth conditions yields a different trend, in order of decreasing sensitivity, Mer, Mcr, HdrDE, Rnf, and Mtr. The sensitivity to Mer is directly due to the fact that the reaction can divert flux away from methane production to CO_2_ production.

Ongoing work on this model aims to test the behavior on other growth substrates such as CO, MMA, DMA and MMS, as well as mixtures of growth substrates. Future work to refine the rate parameter estimates in order to better capture growth defects with gene knockouts of nonessential methanogenesis genes such as heterodisulfide reductase is planned [31].

### 3.5. Transcriptional Regulation Model

#### 3.5.1. Direct Interactions

**Figure 12:**
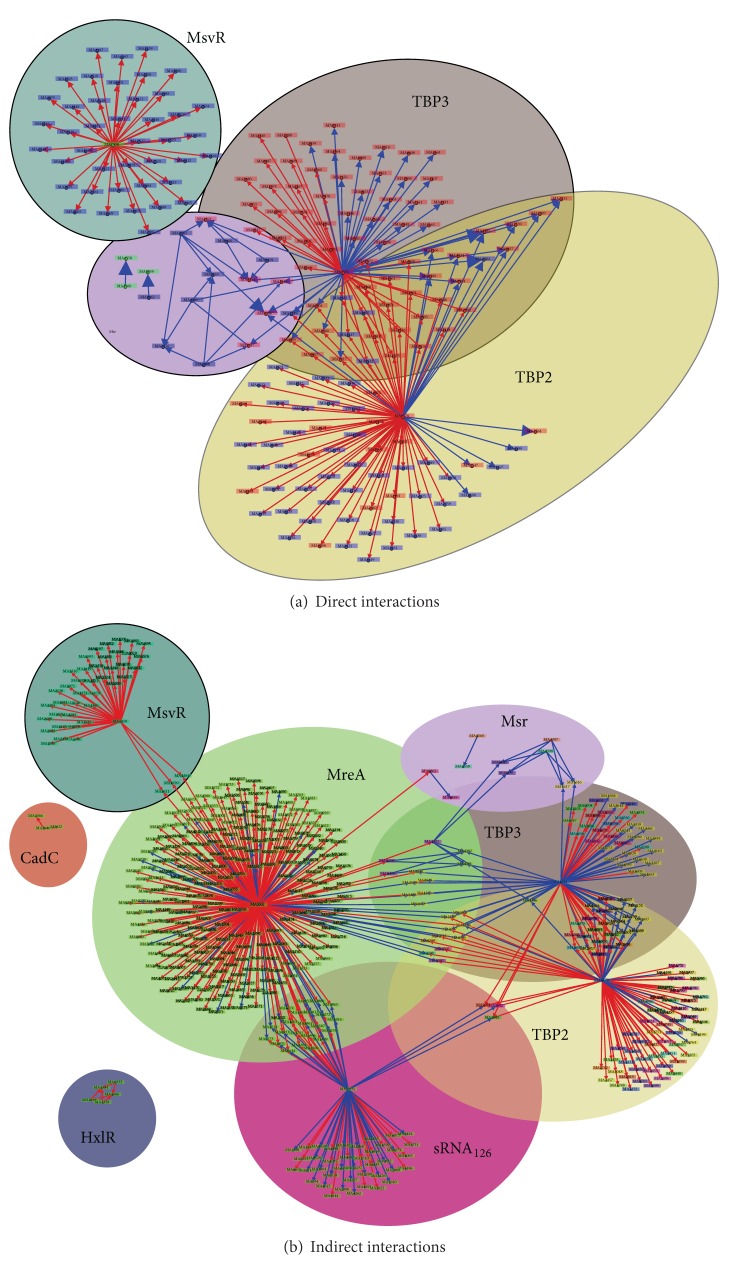
Graph representations of the direct and indirect regulations and associated spheres of influence. Red arrows indicate downregulation and blue arrows indicate up regulation. The regulator name is indicated by the large black text.

The direct interaction map, seen in Figure 12(a), is largely made up of TATA binding proteins (TBPs) which are common across all archaea. Other direct interactions were largely identified only for methyltransferases, nitrogen fixation proteins, and oxidative stress proteins. Three TATA-box binding proteins (TBPs) were identified in *Methanosarcina* spp. and one experiment characterized their role in regulation [72]. While TBP1 is required for growth, and likely the main transcription regulator, TBP2 and TBP3 are dispensable. These two differentially regulate approximately 123 genes on acetate versus methanol growth, and the authors of the study concluded that the two transcription factors optimized protein expression for low-energy substrate (e.g., acetate) growth [72]. These interactions are shown in Figure 12(a).

The second type of direct regulators—those interacting with methyltransferases—act as the mediators for methyl containing organic chemicals entering the methanogenesis pathway. There are separate methyltransferases for each substrate, including methanol, trimethylamine, dimethylamine, monomethylamine, and methylsulfide. Because methyltransferases are some of the most highly expressed genes, they are tightly regulated to preserve the energy balance in the cell [28]. Considerable experimental effort has uncovered eight methyltransferease specific regulators (Msr's). Msr's can act as both up- and downregulators. It was found that in the case of MsrA and MsrB, both proteins act in concert to upregulate expression of MtaCB1, and knockout of either can prevent expression [73]. Similarly, knockout of either *msrD* or *msrE* will prevent expression of MtaCB2 [73]. MsrD and to a lesser extent MsrE also repress MtaCB3 [73]. Some Msr's upregulate one gene, while downregulating other genes; for example, MsrF enhances expression of methylsulfide methyltransferase *mtsD* on all growth substrates except methanol, while MsrC enhances expression of *mtsF* when on all three methylamines [74, 75]. The full set of interactions can be seen in Figure 12(a).

Nitrogen fixation regulation is the third direct set of regulation interactions identified. Two widely conserved nitrogen regulatory proteins named NrpRI and NrpRII have been studied in *Methanosarcinales*. In *M. mazei* Gö1 they were found to regulate 23 proteins that shared a similar DNA regulatory sequence. Overall about 5% of all of *M. mazei* Gö1 genes were regulated under nitrogen limitation, with 83 genes being upregulated [76]. Another study showed that several of the upregulated genes under nitrogen limitation were methylamine specific proteins [77]. However, support for direct regulation of all 83 genes by Nrp regulators was not established, and these connections are instead included in the indirect interaction map. The method of action for repression was shown to be that NrpRI binds the DNA and NrpRII which interacts directly with the TBPs, preventing the RNAP from binding [78]. Importantly, homologs of NrpRI and NrpRII have been identified in *M. acetivorans* that were differentially expressed under nitrogen limiting versus nitrogen sufficient growth [79], and it is likely that these highly conserved (92–94% identical amino acid sequences) regulators have similar function. An additional two small RNA (sRNA) molecules, sRNA_154_ and sRNA_159_ whose function is as of yet unknown, include Nrp binding sites upstream of the start codon [80].

One final set of strongly supported interactions include the repression of certain proteins involved in oxidative stress. As Isom et al. point out [81], the MsvR regulator is homologous to a well characterized variant in *Methanothermobacter thermautotrophicus* and 43 genes in addition to the *msvR* gene in *M. acetivorans* contain the two binding sequences upstream of the TATA box. Their study shows support for a homodimer with cysteine residues which likely are oxidized in an oxygen rich environment, causing the dimer to be released from the binding site.

Overall, the total number of interactions in the direct model is 248, with 10 regulators. Strengths of interaction, where known, are indicated by the width of the arrows in Figure 12.

#### 3.5.2. Indirect/Hypothesized Interactions

Studies of nitrogen-related regulation in *M. mazei* [76] have led to the identification of 69 proteins that were differentially expressed by at least 3-fold [76, 77]. Of the proteins, 35 were involved in nitrogen and energy metabolism, 7 were transport system genes, and 10 were potential regulators. Of particular interest was the upregulation of the *mtb* and *mtm* genes used in methylamine degradation to generate energy or ammonia, the latter of which must be synthesized from N_2_ under starvation conditions. Because many of the identified genes did not have the binding site for the Nrp regulators upstream of their start sites, they are likely regulated by another protein.

One of the more exciting regulations that has been discovered in archaea is an sRNA that targets both *cis-* and *trans-*encoding mRNAs called sRNA_162_ [82]. Overexpression of sRNA_162_ in *M. mazei* greatly upregulated many of the methylamine processing proteins. The work also implicated an ArsR family transcription factor as the mediating component in the regulation [82]. Homologs with high sequence identity (>90%) to both the sRNA and the ArsR regulators (gene MA1531) exist in *M. acetivorans*; therefore, we have included the same interactions in our hypothetical map.

MreA (*Methanosarcina* regulator of energy-converting metabolism) was recently implicated as the global regulator of methanogenesis after it was observed to be 38-fold more highly expressed on acetate than on TMA or methanol [59]. A study comparing the ratio of methanogenesis protein encoding genes in a strain containing a knockout to the wild type indicated that MreA acts to upregulate acetoclastic proteins and downregulate methylotrophic pathways [59], the latter of which is mediated by changes in expression of the Msr proteins previously discussed. Therefore, MreA could act as a switch between methanol and acetate utilization. Adding the interactions reported in the paper to the indirect graph allows MreA to have the greatest putative sphere of influence on gene expression (Figure 12(b)).

Cadmium resistance has been studied in a number of different archaea and bacteria. A well known CadC regulator represses cadmium resistance genes. It is stimulated to unbind by Cd^2+^, Bi^3+^, and Pb^2+^ [83]. This is a particularly interesting regulation as *M. acetivorans* growing on acetate in the presence of cadmium chloride shows between a two- and fivefold increase in methane production, likely attributed to higher levels of acetate kinase and carbonic anhydrase and lower phosphate kinase (Pta) [84]. Furthermore, it has been shown that levels of Coenzyme M increased roughly proportionally to Cd^2+^ concentration [85]. A homolog of the *cadC* gene, MA3940, likely regulates two cadmium efflux encoding genes MA3366 and MA3632 in *M. acetivorans*. In addition, evidence exists for a putative interaction between CadC or one of the genes it regulates and *ack*, *pta*, and carbonic anhydrase. The former two of these interactions are shown in Figure 12(b).

#### 3.5.3. Methanogenesis Gene Regulation

**Figure 13:**
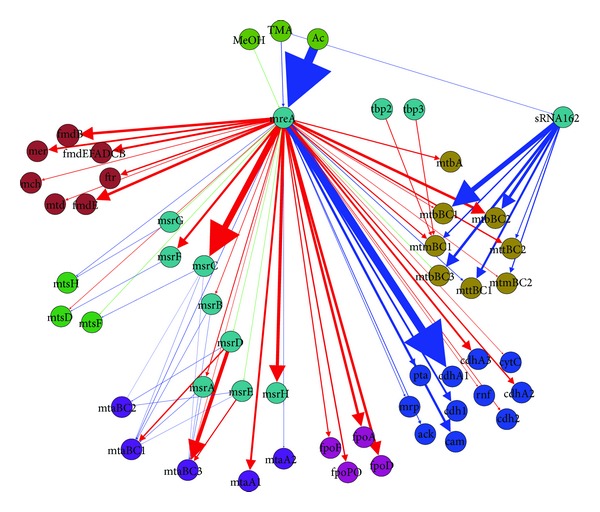
A graph representing the regulation of methanogenesis genes. Red arrows indicate downregulation, blue arrows indicate upregulation, and green arrows indicate weak regulation. The thickness of the arrow shows the strength of the interaction. Turquoise nodes are known regulators. Red nodes are CO_2_ efflux proteins. Olive green nodes are growth substrates. Green nodes are methyl-sulfide methyltransferase proteins. Purple nodes are methanol methyltransferase proteins. Blue nodes are acetate utilization proteins and gold nodes are methylamine methyltransferase proteins.

Simplification of the two regulation maps to only those interactions that directly modulate expression of methanogenesis genes yields the map shown in Figure 13. From this map it can be seen that two regulators (sRNA_162_ and MreA) interact broadly with methanogenesis genes. Correlations of regulator and methanogenesis proteins across several substrates (data not shown) indicate three broad classes of regulation: methanol, acetate, and other methyl-containing substrates (MMA, DMA, TMA, and MMS). Regulation motifs usually embody one of two modes of action in many different species: (1) a global regulator that activates/represses many genes or (2) activation of a single or handful of genes via very specific interaction [86]. Therefore, a possible energetically efficient way to regulate metabolism may be to have a few global regulators that activate/repress many genes, some of which may in turn act as specific regulators capable of fine tuning individual gene expression.

With this knowledge in mind and assuming that the MreA and sRNA_162_ regulators interact in the way proposed in the literature, it can be hypothesized that between the two regulators the three classes of regulation can be covered. In this hypothesis MreA is a global regulator that facilitates the switch between methyl substrates and acetate. It does this primarily by turning off the CO_2_ efflux pathway while turning on the acetate utilization pathway. Upregulation of TMA, DMA, and MMA utilizing proteins is accomplished by expression of the specific regulator sRNA_162_, which turns off expression MA1531, which may be a repressor of methylamine genes.

## 4. Conclusions

We have shown that SiMPull can be used as a method to measure the number of proteins in anaerobic organisms. This entailed genetically engineering a SNAP tag gene into the chromosome along with the gene encoding for the protein of interest, McrG and ribosomal protein Rpl18p. Using this technique we were able to estimate the number of protein complexes in single cells in their exponential growth phase which is important data for modeling. With Mcr's unique position in the methanogenic pathway, knowledge of its copy number is important for modeling metabolic dynamics. As ribosomes are known to be one of the dominant components of molecular crowding, their numbers are important to generate accurate *in silico* whole cell models of methanogens.

Using SiMPull and RNA-seq expression data from monoclonal cell cultures of the methanogens growing on acetate and methanol, we were able to estimate the number of proteins in the methanogenesis pathways. Coupling the resulting cell mass growth reaction to the methanogenesis reactions, we were able to fit unknown rate constants to experiments for growth on methanol. Applying the model to growth on acetate, we were able to capture the correct timescale for use of acetate and production of the methane; however, the cell mass growth rate in exponential phase was too high.

In order to apply the model to more complex scenarios, especially time-varying growth substrate conditions potentially found in the environment, we need a regulation mechanism for expression of the proteins. Examining correlations of protein expression across different substrates leads to the observation that there appear to be three classes of growth: methanol, acetate, and another methyl substrate (TMA, DMA, MMA, and MMS). Towards the goal of developing a regulation model, we have compiled known transcriptional regulation with putative regulation interactions to create a draft model for *M. acetivorans*. Reducing the draft regulation map to just interactions with methanogenesis protein encoding genes, two regulators arise as global regulators. MreA appears to switch between the acetoclastic pathway and the CO_2_ efflux pathway and, therefore, is hypothesized as the switch between acetoclastic growth and methylotrophic growth. sRNA_162_ appears to turn on expression of genes necessary for utilizing methylamines and, therefore, optimizes the organism for methylamine growth.

The physical and stoichiometric properties and kinetic model reported here complement the metabolic reconstructions and constitutes significant progress towards a full computational model of *M. acetivorans*. Spatial heterogeneity, such as that caused by large crowders like the ribosome, is known to cause stochastic effects in similar cells from a monoclonal culture; therefore, quantifying the number and distribution is necessary. Because many reactions in methanogenesis occur in the membrane, stochasticity due to the local environment could have a large effect. Larger spatial organization, such as membrane bound protein complex locality and number, can be determined by cryoelectron tomography. Such data could be used with the kinetic and regulation models developed here to construct detailed full cell reaction-diffusion models similar to those that have been created previously [87]. Such models would allow study of stochasticity in individual organisms. Ultimately, these models could be used with hybrid reaction-diffusion master equation/flux balance analysis techniques [88] that provide full metabolic modeling with spatial effects due to cell culture organization. The utility of the computational models is that they should be easily extendable to the other *Methanosarcina* spp.

## Figures and Tables

**Table 1:** Kinetic model of methanogenesis. Reactions are from *iMB745* [22]. Rate constants used in the kinetic model are taken from the literature where indicated or were fit to experiments of growth on methanol. Water molecules and the intracellular protons (H^+^), which are shown for completeness, are assumed to be constant and are not explicitly modeled. The “Type” column specifies the reaction mechanism: B: irreversible bimolecular Michaelis-Menten, B/C: irreversible bimolecular Michaelis-Menten for the two underlined reactants with a constant flux term for the others, which is set to the flux calculated for bimolecular reaction, M: irreversible unimolecular Michaelis-Menten, F: first order reaction.

Enzyme	Reaction	*k*_cat_ (s^−1^)	*K*_*M*_ (mM)	Type	Citation
Acetoclastic pathway					
Ack	ATP + Ac → AcP + ADP	1055	0.0713	B	[32]
Ack	ADP + AcP → Ac + ATP	1260	0.098	B	[33]
Pta	CoA + AcP → AcCoA + P_i_	1500	0.186	B	[34]
Pta	AcCoA + P_i_ → CoA + AcP	65.8	0.18	B	[35]
Cdh	A cCoA + 2Fd_ox_ + H4SPT + H_2_O → CO + CoA + 2Fd_red_ + MeH4SPT + 2H^+^	358.5	7.1	B/C	[36]
Cdh	CO + CoA + 2Fd_red_ + MeH4SPT + 2H^+^ → AcCoA + 2Fd_ox_ + H4SPT + H_2_O	1130	0.9	B/C	[37]

Methylotrophic pathway					
MtaCBA1	MeOH + CoM + H^+^ → MeCoM + H_2_O	17	50	M	[38, 39]
MtaCBA2	MeOH + CoM + H^+^ → MeCoM + H_2_O	15	50	M	[38, 39]
MtaCBA3	MeOH + CoM + H^+^ → MeCoM + H_2_O	5	50	M	[38, 39]
Mer	MeH4SPT + F_420_ + H^+^ → MethyleneH4SPT + F_420_H_2_	119.7	0.25	B	[40]
Mer	MethyleneH4SPT + F_420_H_2_ → MeH4SPT + F_420_ + H^+^	815	0.3	B	[41]
Mtd	MethyleneH4SPT + F_420_ + 2H^+^ → MethenylH4SPT + F_420_H_2_	2650	0.065	B	[42]
Mtd	MethenylH4SPT + F_420_H_2_ → MethyleneH4SPT + F_420_ + 2H^+^	408	0.065	B	—
Mch	MethenylH4SPT + H_2_O → FormylH4SPT + H^+^	701	0.57	M	[43]
Mch	FormylH4SPT + H^+^ → MethenylH4SPT + H_2_O	100	0.57	M	—
Ftr	FormylH4SPT + Mfr → H4SPT + H^+^ + FormylMfr	1787	0.1	B	[42]
Ftr	H4SPT + H^+^ + FormylMfr → FormylH4SPT + Mfr	262	0.1	B	—
Fmd/Fwd	FormylMfr + 2Fd_ox_ + H_2_O → CO_2_ + 2Fd_red_ + H^+^ + Mfr	1225	0.02	B/C	[44]
Fmd/Fwd	CO_2_ + 2Fd_red_ + H^+^ + Mfr → FormylMfr + 2Fd_ox_ + H_2_O	175	0.02	B/C	—

Shared pathway					
Mtr	H^+^ + MeH4SPT + 2Na_c_^+^ + CoM → H4SPT + 2Na_e_^+^ + MeCoM	50	3.7	B	—
Mtr	H4SPT + 2Na_e_^+^+ MeCoM → H^+^ + MeH4SPT + 2Na_c_^+^ + CoM	50	3.7	B	—
Mcr	MeCoM + CoB → CoBCoM + CH_4_	5.0	2	B	[45]

Electron transport pathway					
HdrDE	CobCoM + MphenH_2_ + 2H^+^ → Cob + CoM + Mphen + 2H_e_^+^	74	0.092	B	[46]
Rnf	2Fd_red_ + 3Na_c_^+^ + Mphen + 2H^+^ → 2Fd_ox_ + 3Na_e_^+^ + MphenH_2_	80	0.1	B/C	—
Fpo	F_420_H_2_ + Mphen + H^+^ → F_420_ + MphenH_2_ + 2H_e_^+^	80	0.1	B	—

Cell growth					
ATP synthase	ADP + P_i_ + 4H_e_^+^ → ATP + H_2_O + 3H^+^	16	0.1	B/C	[47]
Cell Mass^a^	ATP → ADP + P_i_ + Cell mass	0.125^b^	—	F	[22, 31]

^a^The reaction that converts ATP into ADP and cell mass, generating proteins via the stoichiometry in Table 2. ^b^This rate is in units of hr^−1^ which is equivalent to an 8 hr doubling time for *M. acetivorans*.

**Table 2:** A list of enzyme stoichiometries in the cell mass reaction. The moles of the indicated protein that are created from 1 mole of ATP calculated in Section 2.6.

Enzyme	Methanol	Acetate
	(*μ*mol/mol)	(*μ*mol/mol)
Ack	37.5	102.0
ATP	132.0	406
Cdh	151.0	134.4
Fmd/Fwd	57.4	6.4
Fpo	27.8	3.96
Ftr	9.60	4.72
HdrDE	45.3	38.1
Mch	30.0	11.6
Mcr	321.8	398
Mer	25.5	1.26
MtaCBA1	1.97^a^	3.57
MtaCBA2	10.78	5.66
MtaCBA3	0.20^a^	141.0
Mtd	36.9	1.69
Mtr	112.4	144.4
Pta	36.2	171.0
Rnf	22.8	17.1

^a^Expression values of MtaCBA1 and MtaCBA3 were adjusted such that their ratios to MtaCBA2 were in agreement with the protein expression values measured experimentally [30].

**Table 3:** Mean protein copy numbers per cell for Mcr complex and ribosomes as estimated by dividing concentration of McrG and Rpl18p subunits in cell lysates by number of cells in the culture. All experiments were done with three technical replicates and two biological replicates grown in methanol.

Methlyl-coenzyme M-reductase			
Biol.	Conc.	Cells/mL	Count/cell
Rep.	(nM)		
1	1.1 ± 0.13	(5 ± 2) × 10^8^	1320 ± 713
2	0.37 ± 0.03	(8 ± 3) × 10^8^	273 ± 124

Ribosome			
Biol.	Conc.	Cells/mL	Count/cell
Rep.	(nM)		

1	5	(3 ± 1) × 10^8^	10038 ± 3340
2	27.1	(9 ± 3) × 10^8^	18135 ± 6040
